# Transient dataset of household appliances with Intensive switching events

**DOI:** 10.1038/s41597-024-03310-3

**Published:** 2024-05-14

**Authors:** Dongyang Zhang, Xiaohu Zhang, Lei Hua, Jian Di, Wenqing Zhao, Yumei Ma

**Affiliations:** 1 Department of Computer Science North China Electric Power University (Baoding), BaoDing, China; 2 Hebei Key Laboratory of Knowledge Computing for Energy & Power, BaoDing, China; 3 Engineering Research Center for Intelligent Computing of Complex Energy Systems, Ministry of Education, BaoDing, China

**Keywords:** Energy modelling, Energy management

## Abstract

With the development of Non-Intrusive Load Monitoring (NILM), it has become feasible to perform device identification, energy consumption decomposition, and load switching detection using Deep Learning (DL) methods. Similar to other machine learning problems, the research and validation of NILM necessitate substantial data support. Moreover, different regions exhibit distinct characteristics in their electricity environments. Therefore, there is a need to provide open datasets tailored to different regions. In this paper, we introduce the Transient Dataset of Household Appliances with Intensive Switching Events (TDHA^25^). This dataset comprises switch instantaneous data from 10 typical household appliances in China. The TDHA dataset features a high sampling rate, accurate labelling, and realistic representation of actual appliance start-up waveforms. Additionally, appliance switching is achieved through precise control of relay switches, thus mitigating interference caused by mechanical switches. By furnishing such a dataset, we aim not only to enhance the recognition accuracy of existing NILM algorithms but also to facilitate the application of NILM algorithms in regions sharing similar electricity consumption characteristics to those of China.

## Background & Summary

With the rapid advancement of Internet of Things (IoT) technology, there is a growing interest on its application in daily lives, particularly in the flourishing domain of smart home technology. However, the expense associated with implementing smart home solutions has remained a persistent challenge. The emergence of Non-Intrusive Load Monitoring (NILM) presents a promising solution to this issue. NILM technology enables the monitoring of device switches at the main power supply of a household, offering a stark departure from traditional invasive energy monitoring methods that require deploying one sensor per device. This eliminates the need for costly multi-sensor configurations and simplifies installation complexity. Consequently, NILM holds the potential to significantly reduce the overall cost of smart home technology^1,2^.

It is proved that significant reductions in energy waste can be achieved through strategic power-saving practices and management, potentially saving from 5% to 10%. Moreover, the promotion of home energy-saving renovations and efficient operational practices could yield even greater savings, ranging from 10% to 20%^3,4^. Most residential users find it difficult to accurately estimate the energy consumption of household or personal appliances, as indicated by studies. Commonly, residents tend to underestimate energy usage for heating while overestimating consumption from perceptually prominent devices such as lights and televisions. Effective power-saving strategies and retrofitting efforts necessitate a thorough analysis of appliance power load consumption, which in turn, relies on the monitoring and identification of energy usage. Hence, the monitoring on appliance power consumption by NILM is crucial for informed household appliance usage planning and energy consumption reduction^5^.

At present, NILM technologies primarily fall into two categories: event-based detection and appliance energy consumption-based method. Event-based detection focuses on identifying appliance activation and deactivation events, while appliance energy consumption-based approaches concentrate on decomposing energy consumption patterns^6^. Event-based detection technology investigates transient fluctuations in total power states to discern switch activations. Conversely, appliance energy consumption-based methods rely on analysing steady-state characteristics of total power to identify appliance activations through energy consumption decomposition.

Datasets of NILM are typically classified based on their sampling frequencies, with those below and above 1 kHz are considered low and high frequencies, respectively^7^. High-frequency datasets provide more data observation points compared to their low-frequency counterparts, enabling the detection of subtle changes in load waveforms and the identification of additional appliance load characteristics. However, acquiring high-frequency datasets require equipment with higher sampling frequencies, which tends to be more expensive than low-frequency acquisition equipment. Moreover, real-time capabilities and accuracy of the acquisition system is necessary for the high-frequency data acquisition. imposes stricter requirements on real-time capabilities and accuracy of the acquisition system.

The event detection and appliance energy consumption share two fundamental steps: signal measurement and feature extraction. Signal measurement forms the cornerstone of NILM, making publicly available datasets crucial in this field. Such datasets aid researchers in reproducing and refining existing research results, and the quality of the dataset significantly influences the performance of decomposition algorithms^7^. Obtaining data specific to a particular country is essential for testing the performance of algorithms since different countries utilize different appliances and exhibit distinct usage patterns due to cultural variations. Over the past decade, numerous NILM datasets have been released, starting with the pioneering REDD dataset by researchers at MIT in 2011. Subsequently, researchers from various countries including the United States, Canada, India, France, and the United Kingdom have contributed additional datasets. Table 1 summarizes the available information on high-frequency datasets, while Table 2 provides an overview of low-frequency datasets.

**Table 1:** Commonly used high-frequency data sets.

Dataset	Frequency		Duration	Country
	Aggregate	Appliance		
BLUED^16^	12 kHz	N/A	8 days	USA
UK-DALE^17^	16 kHz	1/6 Hz	3–17 months	UK
SustData^18,19^	8 kHz	1 min	5 years	Portugal
WHITED^20^	44.1 kHz	N/A	—	Around the world
COOLL^21^	100 kHz	N/A	6 s	France
PLAID^22^	30 kHz	30 kHz	—	USA
BLOND^23^	50 k-250 kHz	6.4 k-50 kHz	7–32 weeks	Germany
EMBED^24^	12 kHz	1 Hz	27 days	USA
DSUALMH^25^	15.625 kHz	N/A	—	Spain

**Table 2:** Commonly used low-frequency data sets.

Dataset	Frequency		Duration	Country
	Aggregate	Appliance		
REFIT^26^	8 s	8 s	2 years	UK
MEUD^27^	1 min	N/A	1 year	Canada
RAE^28^	1 Hz	N/A	10.3 weeks	Canada
IDEAL^29^	1 Hz	1 Hz	22 months	UK
QUD^30^	3 s to 30 min	3 s to 30 min	1 year	Qatar
IEDL^31^	1 min	1 min	1 year	India

## Methods

### Data acquisition environment

The dataset is collected in a typical family life scenario of China, where the alternating current phase voltage is standardised at 220 volts with a frequency of 50 Hz. The majority of in-home power sources in this region operate on single-phase power. Consequently, the collected dataset primarily consists of data obtained from single-phase power supplies.

### Data acquisition equipment

#### Overall design

**Fig. 1:**
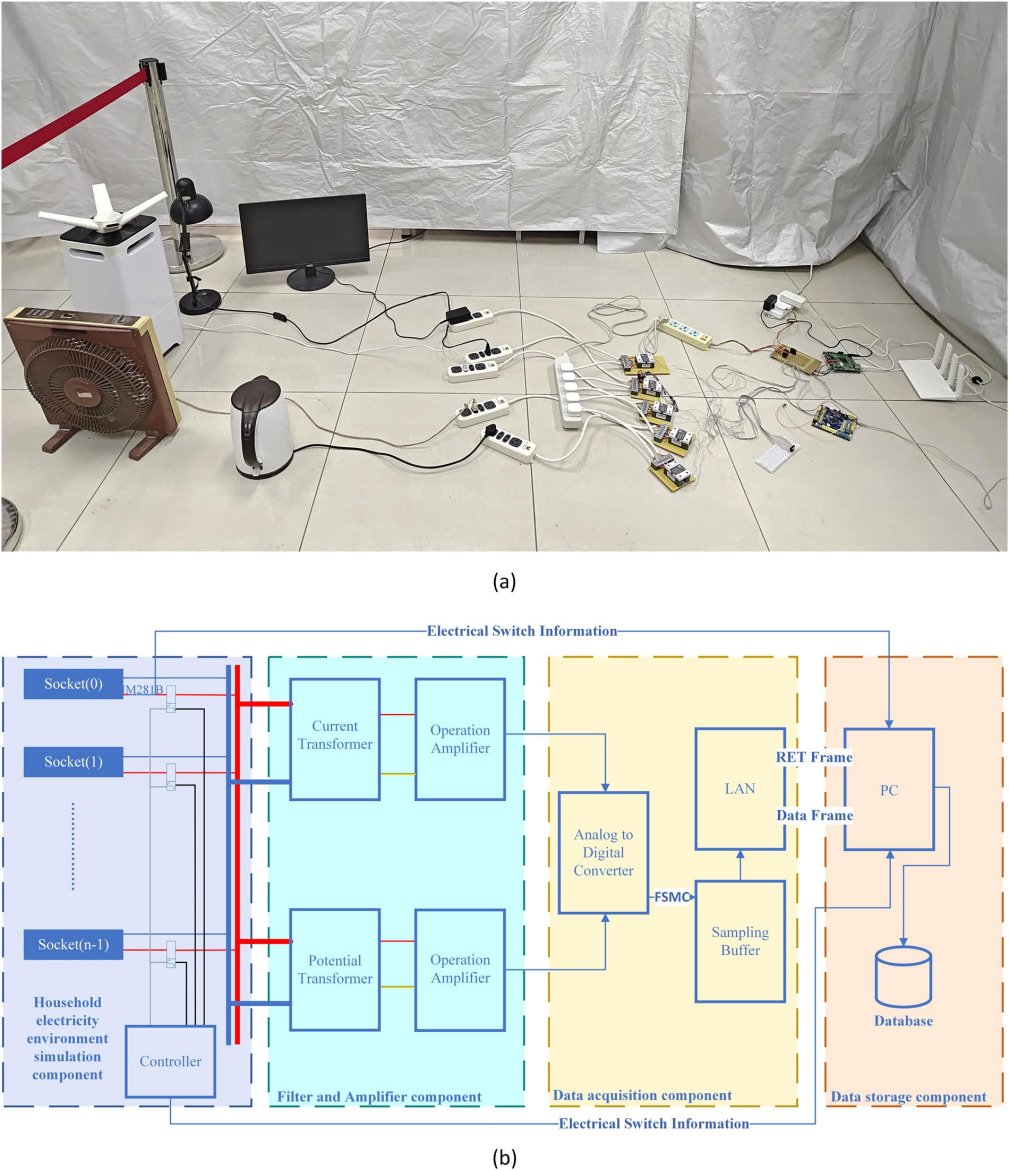
The overall structure of the data acquisition system, (**a**) Component setup for data collection, (**b**) Logic diagram: the system mainly consists of four components (1) Home electricity environment simulation component; (2) Filtering and amplification component (3) Data acquisition component (4) Data storage component.

As depicted in the comprehensive structure of the data acquisition system outlined in Fig. 1, the principal components utilised within the acquisition system are delineated in Table 3. The acquisition device comprises a home electricity environment simulation component, a filtering and amplification component, a data acquisition component, and a data storage component. The details of each component are:

- The home electricity environment simulation component is tasked with replicating the circuit wiring found in a typical home environment. Its primary function is to ensure that the acquired data closely simulates real-world conditions, thus facilitating meaningful comparisons and analyses.
- The filtering and amplification component serves the crucial role of scaling the waveforms of current and voltage from the real environment proportionally to fit within the acquisition range of the ADC chip.
- The data acquisition component is responsible for operating the external ADC chip to precisely sample the current and voltage waveforms. It then transmits the acquired data to the PC, providing accurate raw data for subsequent analysis.
- The data storage component receives data transmitted from the data acquisition component to the PC and archives the collected data into a database, which facilitates subsequent retrieval, analysis, and comparison tasks.

**Table 3:** Information on the main components of the acquisition system.

Hardware type	Hardware Model	Manufacturer
Microcontroller Unit (MCU)	STM32F407IGTx	STMicroelectronics
External ADC	AD7606	Analog Devices Inc. (ADI)
Current Transformers (CT)	SK-MCT224	Shenke (SNK)
Potential Transformer (PT)	SKPT225A-B	Shenke (SNK)
Operational Amplifiers	AD8052	Analog Devices Inc. (ADI)
SRAM	IS61LV51216	Integrated Silicon Solution, Inc. (ISSI)
Ethernet PHY	Ethernet PHY 8720 A	Microchip

Through the seamless integration of these four components, the device is able to effectively simulate the home power environment, precisely collect and securely transmit waveform data of current and voltage.

**Fig. 2:**
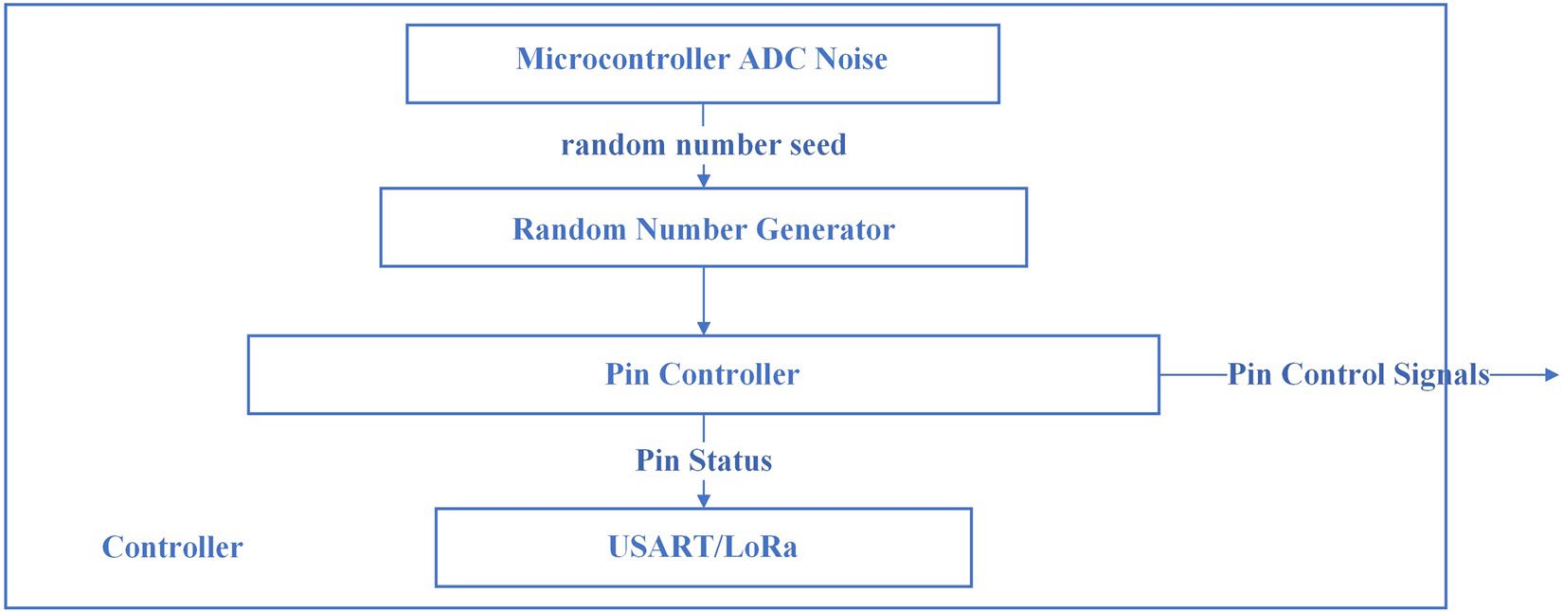
Detailed internal structure of the controller.

In a typical household setting, electricity is distributed to various sockets and appliances throughout the home. To simulate this setup in a laboratory environment, multiple power sockets were utilised to emulate the wiring found in homes. Household appliances are directly plugged into these sockets, while acquisition equipment is connected to the power input of the main socket. As depicted in Fig. 2, each socket on the receptacle is managed by an analogue device switching system comprising a relay and a controller. The relay’s functionality is governed by the output of the controller pins, and the switching data from the relay are transmitted to a computer via a serial port for monitoring and analysis purposes.

**Fig. 3:**
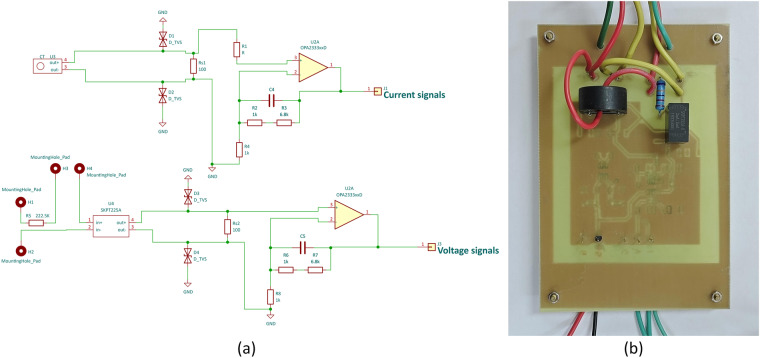
Schematic diagram of filter amplifier circuit.

The filtering and amplification component, as shown in Fig. 3 of the schematic diagram, scales the current to be tested within the range of ±10 V through a current transformer, sampling resistor, and operational amplifier. As the current and voltage waveforms are synchronized, the test voltage, which is scaled to the range of ±10 V, is generated through a potential transformer and operational amplifier. The voltage after amplification is connected to the corresponding pins of an external ADC chip.

**Fig. 4:**
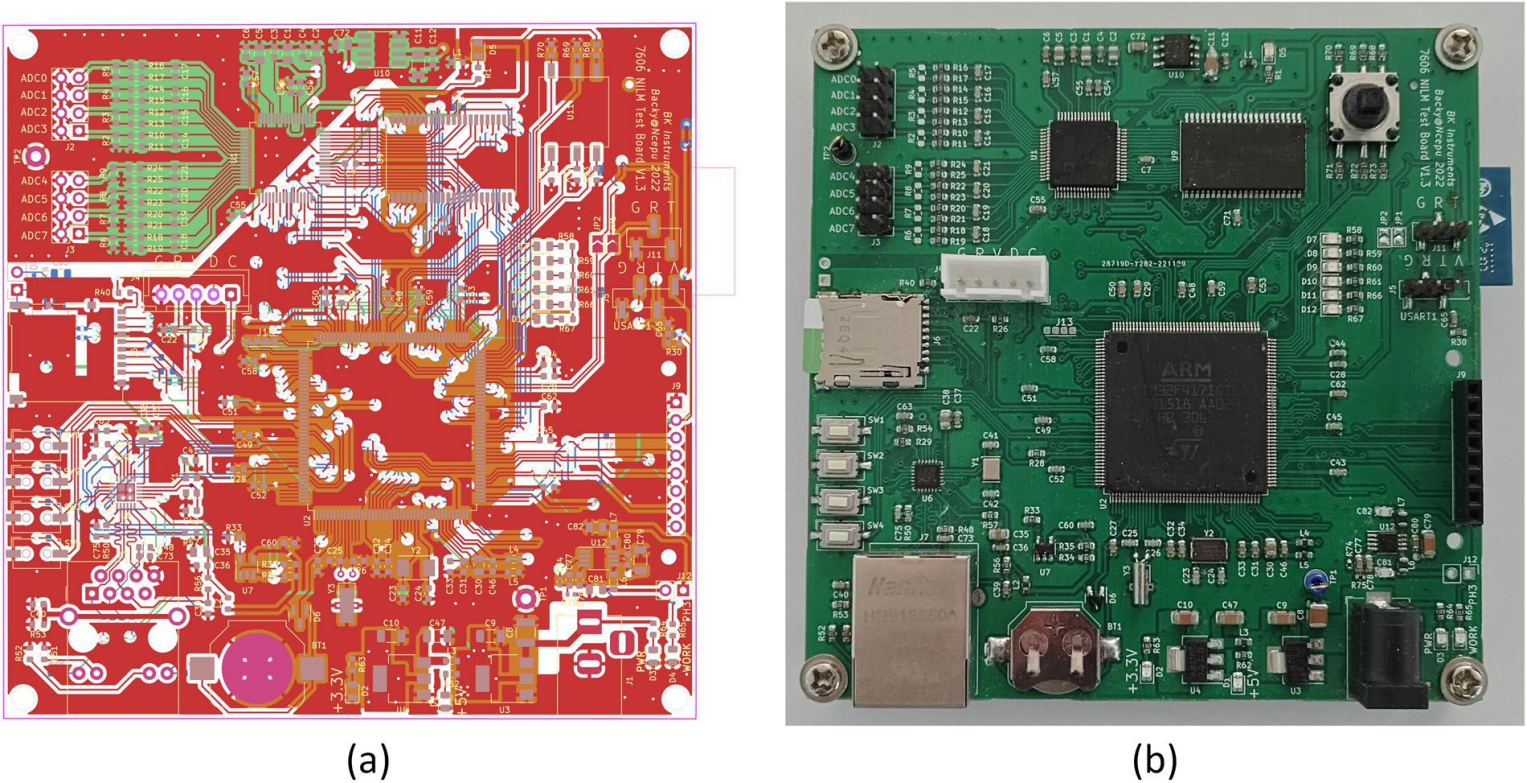
Printed Circuit Board(PCB) of data acquisition circuit.

The data acquisition component, which is depicted in the schematic diagram of the data acquisition circuit in Fig. 4, employs an external ADC chip as an analogue-to-digital converter to facilitate the direct conversion of AC current and voltage waveforms into digital signals. Controlled by the MCU, the external ADC chip reads the data into a buffer, which is then cached through the FSMC using interrupts. This buffered data is subsequently encapsulated into a customised data frame format and transferred to the data storage section via Ethernet utilizing the LWIP protocol (Lightweight TCP/IP Protocol). This framework enables the data acquisition section to efficiently capture current and voltage waveform data and convert it into digital signals for further processing and transmission. The integration of interrupts and buffering mechanisms ensures the accuracy and stability of data collection, while leveraging Ethernet and the LWIP protocol enables rapid data transfer and processing.

**Fig. 5:**
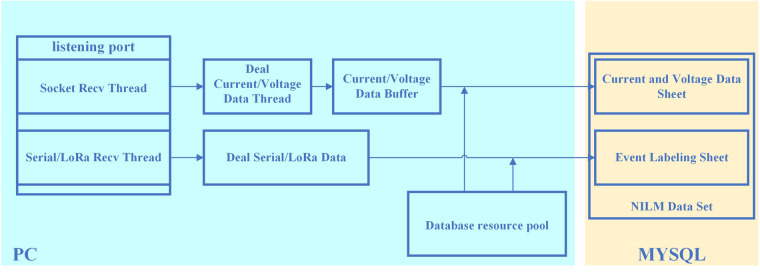
Overall structure of the data storage component.

The data storage component, illustrated in the overall structure of Fig. 5, the establishment of a TCP connection with the data acquisition component by monitoring the corresponding port. Once the connection is established, the data storage module parses the received data frame (as outlined in Table 4) and stores the parsed data in the database. Given that the data acquisition component transmits data every current and voltage cycle (approximately every 20 ms), precautions are taken to prevent potential data loss resulting from the data storage component’s processing speed being lower than the transmission speed of the data acquisition component. To mitigate this risk, the data storage component implements internal buffering, multi-threading, and database resource pool methods to effectively buffer and store the received data. Furthermore, the data storage component is tasked with receiving device switch information from either the serial port or LoRa transmission on the console. These delivery results, along with their corresponding timestamps, are stored in the database.

**Table 4:** Communication frame format diagram.

(a)												
Frame header		Year		Month		Day	Hour		Minute		Second	
32 bit		16 bit		8 bit		8 bit	8 bit		8 bit		8 bit	
**Internal number**		**Room Number**		**Sampling channel**		**Samples per period**	**Sampling value**		**End of frame**		**CRC**	
8 bit		8 bit		8 bit		16 bit	16 bit		16 bit		32 bit	
**(b)**												
**Frame header**	**Internal number**		**Room number**		**Sampling channel**	**Samples per period**		**operation**		**End of frame**		**CRC**
32 bit	8 bit		8 bit		8 bit	16 bit		8 bit		16 bit		32 bit

(a) Data frame format of the data acquisition component and the data storage component (b) Retransmission frame and acknowledgement format of the data acquisition component and the data storage component.

The communication between the data acquisition component and the data storage component involves two distinct frame formats: the data frame outlined in Table 4(a) and the retransmission frame delineated in Table 4(b). These frame formats serve crucial roles in the overall communication process. Firstly, data frames are pivotal in communication as they primarily carry the entirely of actual data acquired from the data acquisition component. It is the responsibility of these data frames to efficiently transfer acquired data to the data storage component for subsequent processing, analysis, and storage. Conversely, retransmission frames serve a different purpose, primarily focusing on ensuring the integrity and reliability of data transmission. In cases of data loss or corruption during communication, the data storage component can utilize retransmission frames to request the retransmission of data from the data acquisition component. This data retransmission mechanism serves to uphold the accuracy and integrity of the transmitted data. The combination of these two frame formats establishes a robust communication framework between the data acquisition and data storage components, aiming to ensure timely data transmission and reliability. Through the synergy of data frames and retransmission frames, the communication system effectively meets the requirements of data acquisition, transmission, and storage, thereby providing a solid foundation for data processing and analysis.

## Data Records

**Fig. 6:**
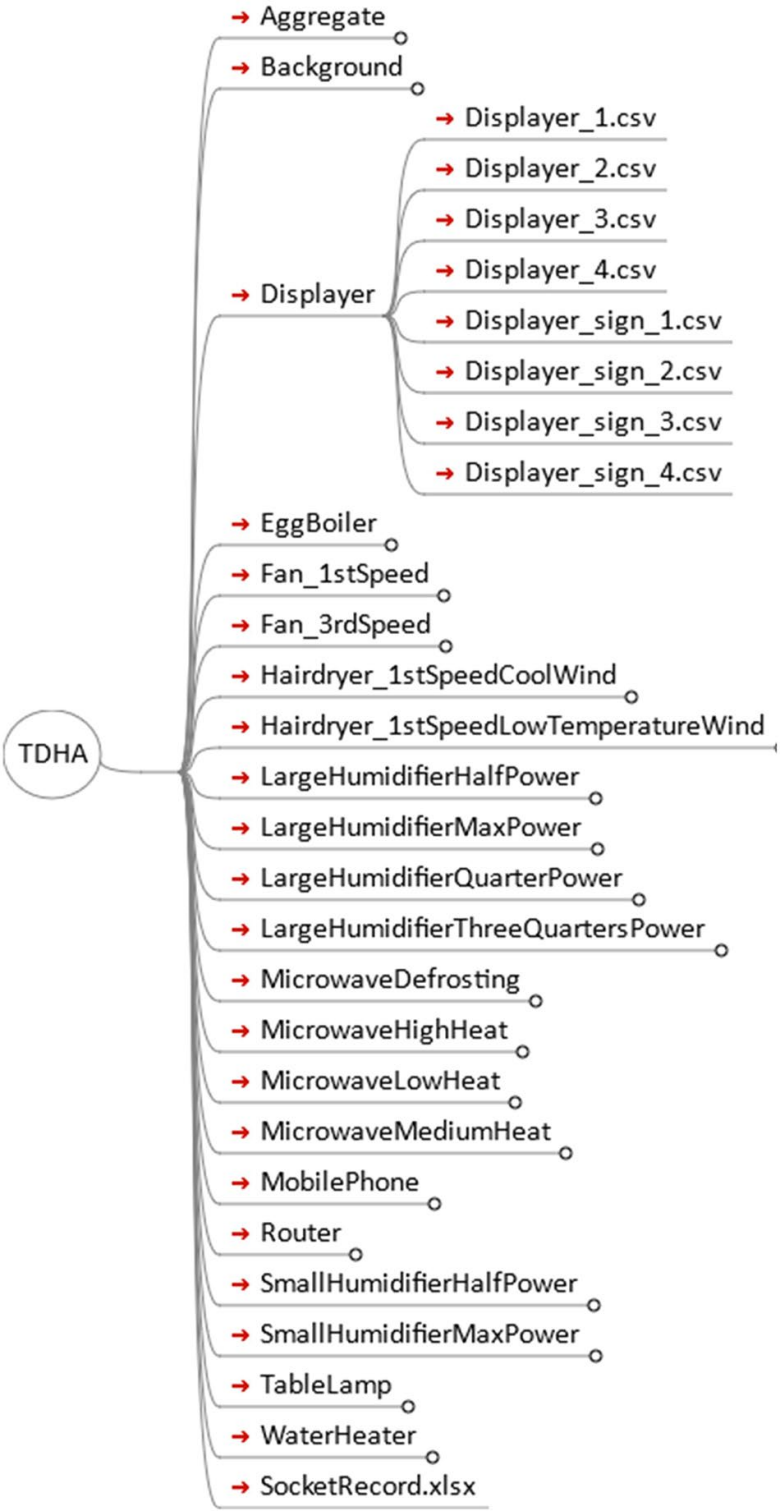
Directory structure of TDHA dataset files.

The TDHA dataset is uploaded to Science Databank (10.57760/sciencedb.13172)^8^. The TDHA dataset consists of 23 files by the time of this paper is published. Its directory structure is shown in Fig. 6.

The Aggregate folder records the instantaneous current and voltage data when the 7 sets of aggregated household appliances are switched on and off, which are stored in separate files named Aggregation_N.csv (N = {1,2…7}), respectively. The labelling of the switching times of these seven sets of aggregated household appliances is stored in the Event folder.

The SocketRecord.xlsx file records information about the appliances that were accessed during the measurement of the 7 sets of aggregated appliance data. This file contains 7 worksheets, each of which is corresponding to a set of aggregated household appliance data.

The Background folder records background current and voltage data in the absence of household appliances being connected. It is mainly used to record the background noise of current and voltage in the absence of household appliances. The folder contains two files: background_5Relay.csv and background_NoRelay.csv.

- The background_5Relay.csv file records the data in the case where there are no household appliances connected and only relays are connected.
- The background_NoRelay.csv file records the data in the case where there is no household appliance access and no relay access.

The remaining folders record instantaneous current and voltage data for various household appliances when switched individually in different on/off states. The names of these folders are a combination of the name of the household appliance and the setting (if the appliance has only one setting, the folder name is the name of the household appliance). Take the folder named “Displayer” as an example:

- Displayer_N.csv (N = {1,2…7}): Records the instantaneous current and voltage data file when the displayer is switched on/off individually.
- Displayer_sign_N.csv (N = {1,2…7}): A labelled file that records the switching time of the displayer.

**Fig. 7:**
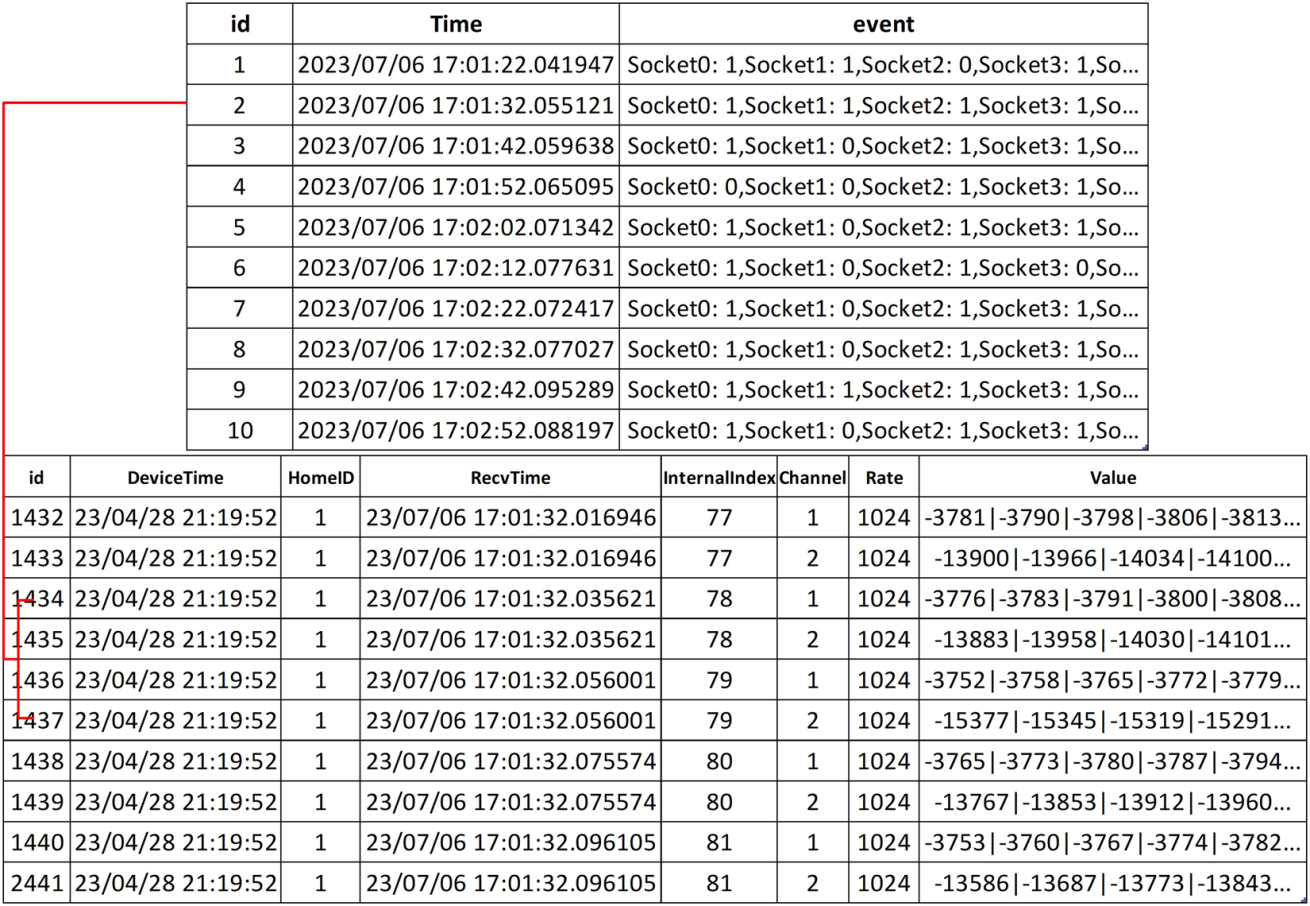
File format diagram for data files.

For data files (such as Displayer_1.csv), each record represents one cycle (20 ms) of current and voltage, as depicted in Fig. 7. Each record includes the raw values (Value) of 1024 data points collected for the current and voltage within that cycle. Additionally, the records contain timestamp markers (RecvTime) shared by the collection and labelling system. The remaining columns are the number of sampling points per cycle (Rate = 1024), the sampled channel (Channel = 1 for voltage, Channel = 2 for current), the room identifier (HomeID, which is a simulated household electricity environment number based on the setup), and the microcontroller RTC time (DeviceTime, this attribute holds no specific meaning and is solely used to check the integrity of the data files).

The annotations for the dataset are stored in other CSV files, such as Displayer_sign_1.csv. Whether it’s for multi-device measurements or single-device measurements, the format of the annotation file remains consistent, as shown in Table 5. Each record in the file consists of a system-level timestamp (RecvTime, timestamp accurate to milliseconds) and a device switching event (event, for individual device labelling format: room number - appliance switch; for aggregated data labelling format: socketx:0/1). Due to the differences in transmission speeds, the annotation times in these files may experience a delay of 20 ms approximately after the appliances are activated.

**Table 5:** Annotation file format diagram with (a) event annotations for single appliance switch measurements and (b) event annotations for aggregated switch measurements for multiple appliances randomly opening and closing.

(a)		
id	Time	Event
1	2023/05/26 10:06:01.721198	1-0
2	2023/05/26 10:06:11.717067	1-1
3	2023/05/26 10:06:21.726447	1-0
4	2023/05/26 10:06:31.724428	1-1
5	2023/05/26 10:06:41.723452	1-0
6	2023/05/26 10:06:51.722633	1-1
7	2023/05/26 10:07:01.726876	1-0
8	2023/05/26 10:07:11.718271	1-1
9	2023/05/26 10:07:21.721194	1-0
10	2023/05/26 10:07:31.728876	1-1
**(b)**		
1	2023/07/06 17:01:22.041947	Socket0: 1,Socket1: 1,Socket2: 0,Socket3: 1,So…
2	2023/07/06 17:01:32.055121	Socket0: 1,Socket1: 1,Socket2: 1,Socket3: 1,So…
3	2023/07/06 17:01:42.059638	Socket0: 1,Socket1: 0,Socket2: 1,Socket3: 1,So…
4	2023/07/06 17:01:52.065095	Socket0: 0,Socket1: 0,Socket2: 1,Socket3: 1,So…
5	2023/07/06 17:02:02.071342	Socket0: 1,Socket1: 0,Socket2: 1,Socket3: 1,So…
6	2023/07/06 17:02:12.077631	Socket0: 1,Socket1: 0,Socket2: 1,Socket3: 0,So…
7	2023/07/06 17:02:22.072417	Socket0: 1,Socket1: 0,Socket2: 1,Socket3: 1,So…
8	2023/07/06 17:02:32.077027	Socket0: 1,Socket1: 0,Socket2: 1,Socket3: 1,So…
9	2023/07/06 17:02:42.095289	Socket0: 1,Socket1: 1,Socket2: 1,Socket3: 1,So…
10	2023/07/06 17:02:52.088197	Socket0: 1,Socket1: 0,Socket2: 1,Socket3: 1,So…

The dataset primarily includes high-sampling-rate raw voltage and current waveforms from household electrical circuits and appliances. It also encompasses voltage and current waveforms of the same appliance under various operating conditions, as well as during random on/off transitions. Additionally, it contains voltage and current waveforms when no appliances are connected to the electrical circuit.

To facilitate waveform analysis, the sampling frequency for both current and voltage waveforms is set to 51.2 kHz, resulting in 1024 samples per cycle for each current and voltage waveform. Additionally, household appliances are systematically switched on and off at regular intervals of every 10 seconds, ensuring a high information density in the dataset.

NILM has categorised appliances into four types based on the nature of their operation^9^:

- Type I: Appliances with only two operating states (on/off) such as cell phone chargers, incandescent lamps, etc.
- Type II: Multi-state appliances with a limited number of operating states, e.g., hair dryers, electric drills, etc.
- Type III: Appliances with continuously variable operating states with a variable number of states, e.g., humidifiers, stereos, etc.
- Type VI: Devices that operate in a constant number of states over a period of weeks or days, e.g., routers, refrigerators, etc.

### Raw voltage and current waveforms of household circuits and appliances

**Fig. 8:**
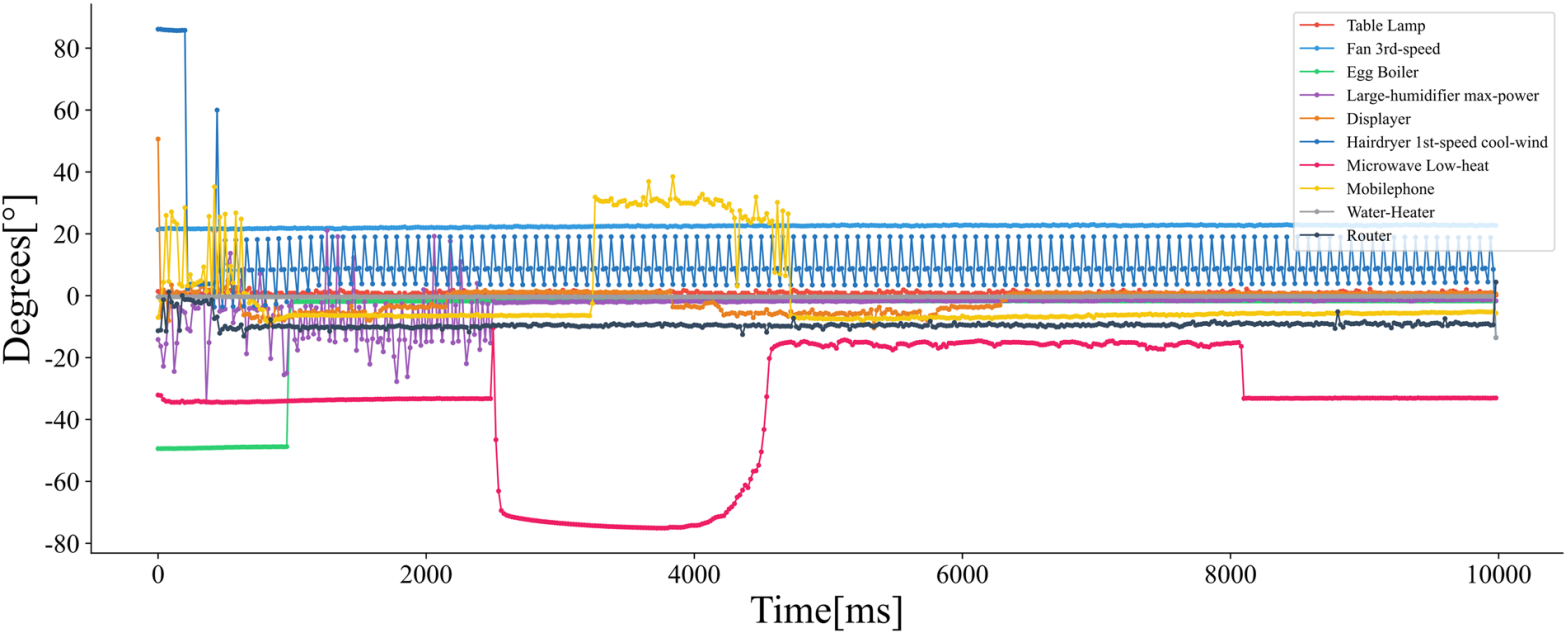
Appliance load type chart, with the load states (capacitive, inductive and resistive loads) presented by some household appliances at the moment of start-up labeled by phase differences.

We compiled a list of common household appliances typically found in Chinese households and meticulously recorded the switching events of each appliance individually. This was accomplished using a data acquisition system alongside an analogue equipment switching system. Table 6 provides a detailed description of the appliances utilised in the setup. Additionally, we categorized these appliances based on their characteristics, classifying them into capacitive, inductive, and resistive loads. Each type of load serves a distinct role in electrical circuits, with their phase difference characteristics enable the distinction among the types of appliances load for the researchers^10^. Figure 8 illustrates the load types of the appliances used in our simulated environment and shows their phase differences.

**Table 6:** List of household appliances used in the TDHA dataset, the table provides the brand, rated power, device type, and load type of the household appliances.

Serial number	Device type	Brand	Number	Rated power	State type	Load type
1	Table Lamp	FSL	1	25 W	Type I	Resistive loads
2	Mobile Phone	VIVO	1	33 W	Type I	Capacitive loads
3	Display	AOC	1	—	Type I	Inductive loads
4	Large Humidifier Quarter Power	RONG SHENG	1	50 W	Type III	Inductive loads
	Large Humidifier Half Power					Inductive loads
	Large Humidifier Three Quarters Power					Inductive loads
	Large Humidifier Max Power					Inductive loads
5	Small Humidifier Half Power	Midea	1		Type III	Capacitive loads
	Small Humidifier Max Power					Capacitive loads
6	Fan 3rd-speed	——	1	30 W	Type II	Capacitive load
	Fan 1st-speed					Inductive load
7	Electric Kettle	Midea	1	1800W	Type I	Resistive loads
8	Router	MIUI	1	20 W	Type VI	Inductive load
9	Hairdryer 1st-Speed Cool Wind	MIUI	1	1800W	Type II	Capacitive load
	Hairdryer 1st-Speed Low Temperature Wind					Capacitive load
10	Microwave Defrosting	Midea	1	600W-900W	Type II	Inductive loads
	Microwave High Heat					Inductive loads
	Microwave Low Heat					Inductive loads
	Microwave Medium Heat					Inductive loads
11	Egg Boiler	Joyoung	1	360 W	Type I	Resistive loads

### Current and voltage waveforms of the same appliance under different operating conditions

**Fig. 9:**
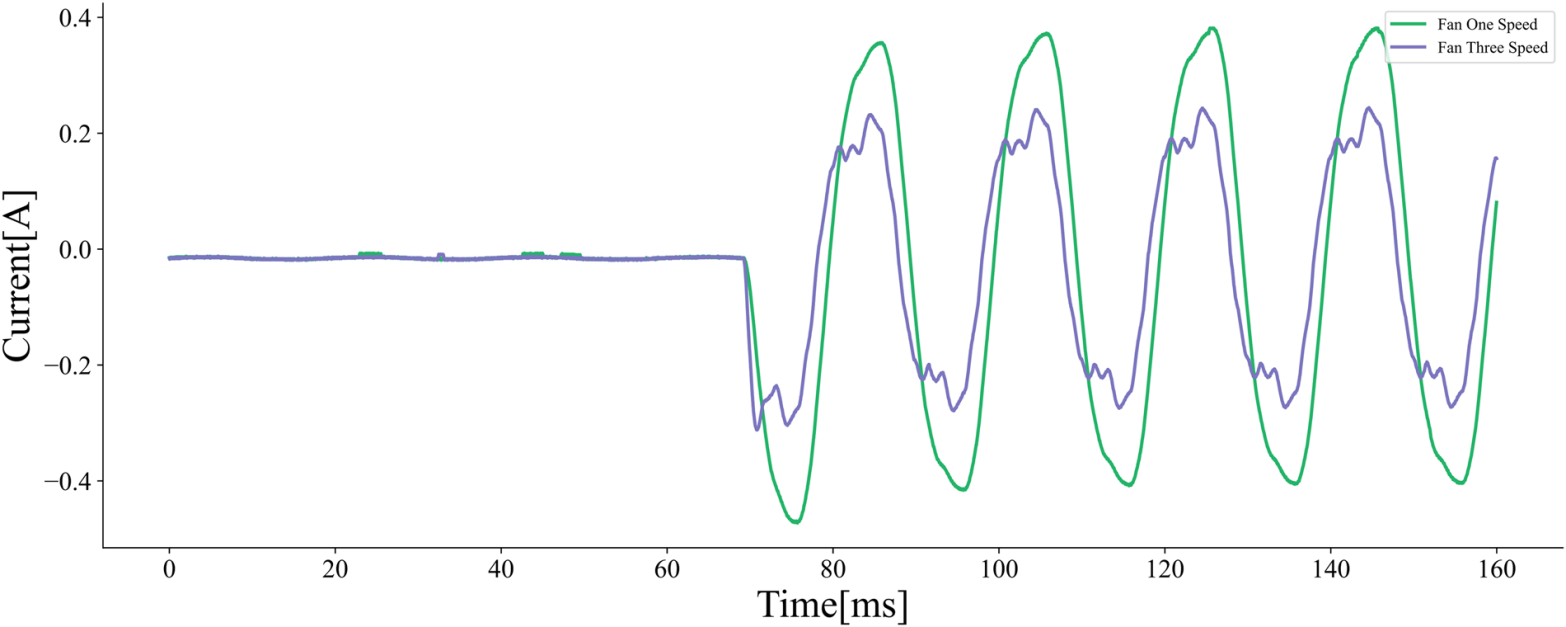
Comparison of different settings of the fan, Starting current waveforms of the fan in first and third speed level.

Based on the classification of appliance operation characteristics, Type I appliances exhibit only two operating states, requiring consideration of just one state. Conversely, Type II and Type III appliances, such as humidifiers and variable-speed fans, operate in multiple distinct modes. We collected current and voltage waveforms for these two types of appliances across various operational states, aiming to improve identification accuracy. Figure 9 illustrates the current and voltage waveforms of a fan starting in first and third level of speed.

### Current-voltage waveforms at random switch of household appliances

**Fig. 10:**
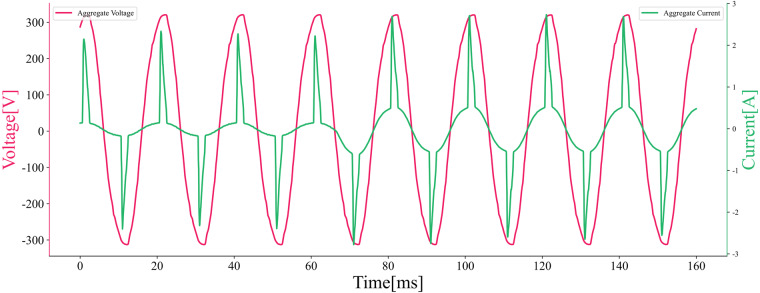
Aggregate current vs. voltage plot, shown at Aggregate 1 (Socket0: 1,Socket1: 1,Socket2: 1,Socket3: 0,Socket4: 0 → Socket0: 1,Socket1: 1,Socket2: 1 Socket0: 1,Socket1:1,Socket2:1, Socket2:1).

This segment of data collection requires the use of a home environment simulation component, which is used to simulate the on/off states of household appliances in a home environment. For the simulation of equipment switch, the household appliances keep their behaviour unchanged during the operations of other existing appliances, i.e., the appliances operate independently. For smart devices whose operating states cannot be directly controlled by relays, we use power metering modules to measure such devices, and a jump in the measured current value indicates that the device is turned on or off. Table 7 shows the appliances used in the simulated aggregated home environment. The current waveforms of an aggregated appliance at a given time of appliance switching are illustrated in Fig. 10.

**Table 7:** Information on appliances used in the simulated home environment.

The electrical appliances connected to the power strip in aggregated data 1						
Socket Device	0	1	2	3	4	Notes
Table Lamp	√					
Mobilephone		√				
Displayer						
Large humidifier			√			Quarter Power
Small humidifier						
Water Heater				√		
Fan					√	1st Speed
Hairdryer						
Router						
Egg Boiler						
Microwave						
**The electrical appliances connected to the power strip in aggregated data 7**						
Table Lamp						
Mobilephone						
Displayer						
Large humidifier		√				Three Quarters Power
Small humidifier	√					Max Power
Water Heater						
Fan			√			1st Speed
Hairdryer						
Router						
Egg Boiler				√		
Microwave					√	High Heat

## Technical Validation

### Data storage

Due to the high sampling frequency of the data collection equipment, a large volume of data is generated within a short period. Therefore, it is essential to minimize the generation of unnecessary datasets. Experimental results based on the setup indicate that the input current and voltage of most appliances remain stable within a 10-second interval. Hence, we regulated the switching of appliances within a 10-second timeframe. As a result, there are 360 appliance switch events per hour. The dataset does not account for user usage patterns and the collected data is not continuous. Instead, it focuses mainly on identifying appliances based on their intrinsic characteristics, by which the generalizability of the dataset is enhanced.

### Data accuracy

The voltage and current transformers, along with the operational amplifiers used in the filtering and amplification section, possess the following characteristics:

Potential transformer

$$Primary\,rated\,current\left(Ib\right)=2mA,$$

$$Secondary\,rated\,current=2mA,$$

$$Secondary\,load=80\Omega $$

$$Linearity\ge 99.6{\rm{ \% ,}}\,{\rm{and}}$$

$$12{\prime} \le Phase\,Difference\le 19{\prime} $$

Current transformers

$$Primary\,rated\,current\left(Ib\right)=5A,$$

$$Secondary\,rated\,current=2mA,$$

$$Secondary\,load=10\Omega ,$$

$$CT\,transformation\,ratio=\frac{5A}{2mA}=2500,$$

$$Linearity\ge 99.8 \% ,$$

$$Phase\,Difference\le 15{\prime} ,$$

Operational amplifiers

$$High\,slew\,rate=145V/\mu s,$$

$$Linearity\ge 99.91 \% ,$$

$$Low\,offset\,voltage\,drift=10\,\mu V{/}^{\circ }C,$$

The ADC chip used for data set acquisition is the AD7606, its characteristics under ±10V acquisition conditions are shown in Table 8, which is set to oversample the ADC chip twice, and the data set is sampled at a frequency of is sampled at a frequency of 51.2 kSPS. The ADC has the following characteristics:

$$Resolution=16$$

$$SN{R}_{k}=90;k=No\,oversampling;\pm 10\,V\,range;{f}_{IN}=1kHz,$$

$$SN{R}_{k}=95.5;k=Oversampling\,by\,16;\pm 10\,V\,range;{f}_{IN}=130\,Hz,$$

$$Linearity=99.9848{\rm{ \% ,}}$$

$$Conversion\,Time=4us,$$

**Table 8:** Transmission Characteristics of the AD7606 with a Sampling Range of ±10 V.

	+FS	MIDSCALE	–FS	LSB
±10 V RANGE	+10 V	0 V	–10 V	305μV

The overall linearity of the acquisition device is then:

$$Linearit{y}_{I-tot}\ge Linearit{y}_{CT}\ast Linearit{y}_{OA}\ast Linearit{y}_{ADC}=99.8{\rm{ \% }}\ast 99.91{\rm{ \% }}\ast 99.9848{\rm{ \% }}\approx 99.6950{\rm{ \% }}$$

$$Linearit{y}_{U-tot}\ge Linearit{y}_{PT}\ast Linearit{y}_{OA}\ast Linearit{y}_{ADC}=99.6{\rm{ \% }}\ast 99.91{\rm{ \% }}\ast 99.9848{\rm{ \% }}\approx 99.4952{\rm{ \% ,}}$$

Because the acquisition device has a linearity of up to 99.4952% and 99. 6950% for voltage and current, respectively, the acquisition device is able to accurately capture subtle signal changes in voltage and current.

The correspondence between the ADC chip sampling value and the actual value is shown in Eq. (1)

1 $$VIN=\begin{array}{c}REF=2.5,\\ \frac{ADRange\ast ADC\,CODE\ast 2.5}{{2}^{15}* REF}\end{array}$$

### Integrity detection

#### Transport integrity

In order to ensure the stability of data transmission, the data acquisition component of the data acquisition system adopts the lightweight TCP/IP (LWIP) protocol, the staging buffer, CRC checksum, and retransmission mechanism. The LWIP protocol is mainly responsible for sending the data frames, and at the same time detecting whether the data are sent successfully. The CRC checksum is mainly responsible for checking the data frames to ensure the accuracy of data transmission. The buffer temporarily stores the data that have been sent and deletes the corresponding records from the temporary storage area upon receiving the confirmation frame for the received data. The retransmission mechanism retransmits the corresponding data frames through the staging buffer when LWIP detects a transmission failure or a CRC check error. If the retransmission fails three times, the retransmitted data frames are stored in the SD card and marked in the LWIP transmission log. Meanwhile, the data acquisition component detecting whether the TCP connection is disconnected, and attempting to re-establish a connection with the data storage buffer if a disconnection is detected. After the data acquisition section finishes running, the failed data is manually written to the database by the SD card.

#### Document integrity

**Fig. 11:**
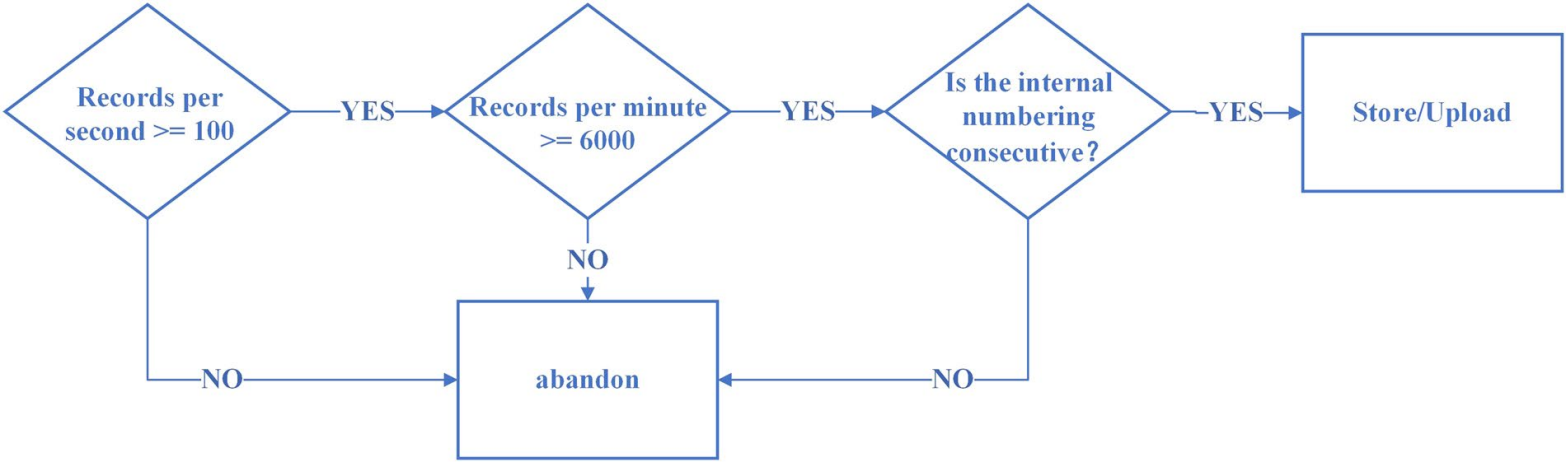
File Integrity Detection Flowchart.

Before uploading the dataset to the website, we have checked each dataset file in detail to make sure that the dataset uploaded to the website have no missing records due to perturbations in the collection process. The integrity checking process is shown in Fig. 11. First, the number of records per second is checked as the calculation of the number of dual-channel records per second should be greater than or equal to 100 records per second, the number of records per minute is greater than or equal to 6000 records. At the same time, we also check whether the internal numbering in each second is continuous.

## Usage Notes

This dataset is provided by CSV files which contains two formats of CSV files as raw dataset waveform file format and event annotation file format, respectively, which can be extracted by using common programming languages and libraries (e.g. Python, MATLAB, etc.). The V2 version of the dataset presented in this paper is released in 2023. The types of appliances, time of collection, amount of data, and the size of aggregated data in this dataset keep updating and releasing over time.

The waveform of current and voltage in this dataset is the original data collected by ADC without any processing, if it is necessary to convert the raw data into actual current and voltage data, he/she need to map the original data, and it is recommended to refer to Eqs. (2, 3):

2 $$\begin{array}{l}VIN=\frac{Actual\,Current}{CT\,transformation\,ratio}\ast {R}_{s}1\ast \left(1+\frac{{R}_{2}+{R}_{3}}{{R}_{4}}\right)\ast Linearit{y}_{I-tot}=\frac{ADRange\ast Original\,Current\,Value}{{2}^{15}}\\ \begin{array}{c}Actual\,Current=\frac{ADRange\ast Original\,Current\,Value\ast {R}_{4}}{Linearit{y}_{I-tot}\left[{R}_{s}1\ast \left({R}_{2}+{R}_{3}+{R}_{4}\right)\right]\ast CT\,transformation\,ratio\ast {2}^{15}}\\ =\frac{{\rm{Original}}\,{\rm{Current}}\,{\rm{Value}}}{{2}^{15}}\ast 28.47\end{array}\end{array}$$

3 $$\begin{array}{l}VIN=\frac{Actual\,Voltage}{{R}_{5}}\ast {R}_{s}2\ast \left(1+\frac{{R}_{6}+{R}_{7}}{{R}_{8}}\right)\ast Linearit{y}_{U-tot}=\frac{ADRange\ast Original\,Voltage\,Value}{{2}^{15}}\\ \\ \begin{array}{lll}Actual\,Voltage & = & \frac{ADRange\ast Original\,Voltage\,Value\ast {R}_{5}\ast {R}_{8}}{Linearit{y}_{U-tot}\left[{R}_{s}2\ast \left({R}_{6}+{R}_{7}+{R}_{8}\right)\right]\ast {2}^{15}}\\  & = & \frac{Original\,Voltage\,Value}{12.90}\end{array}\end{array}$$

**Fig. 12:**
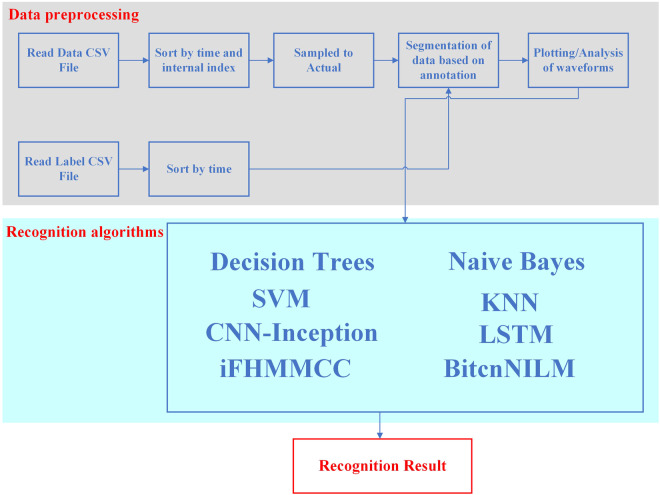
Flowchart of the overall use of the dataset.

The overall flow of using the dataset is shown in Fig. 12. Starting with reading all the CSV files, the data files and labelling files are sorted with respect to their time and internal indexes. Then, the sampled values in the data files are converted to real values according to Eqs. (2) and (3). Subsequently, the data file is segmented according to the time information in the labelled file. By processing the segmented data, the waveforms of the current and voltage can be plotted or analysed using a programming language such as Python or MATLAB. Further, recognition algorithms can be designed and recognition models can be trained^11^, such as Decision Trees^7^, Naive Bayes, Support Vector Machine (SVM), K-Nearest Neighbors (KNN)^7^, infinite factorial Hidden Markov Model (iFHMMCC)^12^, Long Short-Term Memory (LSTM) network^13^, Sequential Point Learning Algorithm with Bidirectional Expansion Convolution (BitcnNILM)^14^, and inception structure algorithm of multiple overlapping sliding windows combined with CNNs^15^ to obtain the final recognition results.

Known issues

- For combinations of multiple household appliances, there are various types of combinations. This dataset only collects data for instances where one combination of household appliances is activated at a time.
- Due to the difference in transmission rates between electrical signals and marker information, there is an approximate deviation of one current-voltage cycle (20 ms) in the timestamps of marked household appliance switch events.

## Data Availability

We used Python to write programmes to process and validate the dataset. Here are some of the programmes we used: • Check.py: This programme is used to validate and check the accuracy of the dataset. • ShowWave.py: This file is used to view the waveform of the current when the appliance is started. • ShowWaveFFT.py: This file is used to perform a Fast Fourier Transform on the current and voltage waveforms. • LWIP_NILMF417IGT_MakeFile_CRC_new: This folder contains the embedded programme used in the data acquisition section. • Upper_computer_monitoring_system.mp4: This file is the video of the monitoring programme of the upper computer during the aggregation data collection. These programmes are helpful in efficiently processing and validating the datasets to ensure the correctness and usability of the data. The source code of this programme has been posted on https://github.com/TagEnd/TDHA-Acquisition-System-Submit and the TDHA dataset has been posted both on the Science Databank https://www.scidb.cn/en/detail?dataSetId=876623ff38634ccb8426b07146720914&version=V2 and on our custom platform http://f-lab.ncepu.edu.cn/TDHA.
